# The Biocontrol Functions of *Bacillus velezensis* Strain Bv-25 Against *Meloidogyne incognita*

**DOI:** 10.3389/fmicb.2022.843041

**Published:** 2022-04-07

**Authors:** Xue-liang Tian, Xiao-man Zhao, Song-yu Zhao, Jian-long Zhao, Zhen-chuan Mao

**Affiliations:** ^1^Henan Engineering Research Center of Biological Pesticide & Fertilizer Development and Synergistic Application, Henan Institute of Science and Technology, Xinxiang, China; ^2^Insititute of Vegetables and Flowers, Chinese Academy of Agricultural Sciences, Beijing, China

**Keywords:** *Bacillus velezensi*s, *Meloidogyne incognita*, fumigation, induced resistance, biological control

## Abstract

*Meloidogyne incognita* is obligate parasitic nematode with a wide variety of hosts that causes huge economic losses every year. In an effort to identify novel bacterial biocontrols against *M. incognita*, the nematicidal activity of *Bacillus velezensis* strain Bv-25 obtained from cucumber rhizosphere soil was measured. Strain Bv-25 could inhibit the egg hatching of *M. incognita* and had strong nematicidal activity, with the mortality rate of second-stage *M. incognita* juveniles (J2s) at 100% within 12 h of exposure to Bv-25 fermentation broth. The *M. incognita* genes *ord-1*, *mpk-1*, and *flp-18* were suppressed by Bv-25 fumigation treatment after 48 h. Strain Bv-25 could colonize cucumber roots, with 5.94 × 10^7^ colony-forming units/g attached within 24 h, effectively reducing the infection rate with J2s by 98.6%. The bacteria up-regulated the expression levels of cucumber defense response genes *pr1*, *pr3*, and *lox1* and induced resistance to *M. incognita* in split-root trials. Potted trials showed that Bv-25 reduced cucumber root knots by 73.8%. The field experiment demonstrated that disease index was reduced by 61.6%, cucumber height increased by 14.4%, and yield increased by 36.5% in Bv-25–treated plants compared with control. To summarize, *B. velezensis* strain Bv-25 strain has good potential to control root-knot nematodes both when colonizing the plant roots and through its volatile compounds.

## Introduction

Globally, annual losses caused by plant parasitic nematodes are estimated at approximately US $157 billion (Abad et al., 2008), of which more than half are due to the root-knot nematode (RKN; *Meloidogyne* spp.) (Kim et al., 2016). RKN infects almost all cultivated plants (Trudgill and Blok, 2001), including vegetables, beans, grains, grass shrubs, fruit trees, and woody ornamental plants (Bagheri et al., 2014). Among *Meloidogyne* spp., *Meloidogyne incognita* is the most destructive because of its short generation time, high reproduction rate, and ability to form complex diseases with other soil-borne pathogens (Vos et al., 2013). Chemical nematicides, such as thiazophos, methamecyl, and abamectin, have been widely used for effective control against RKNs (Giannakou and Karpouzas, 2003); however, they can be highly toxic to animals, humans, and the environment. In order to maintain ecological balance, looking for other viable alternatives, such as biocontrol methods, is seen as one of the primary goals of future nematology (Javed et al., 2012).

In the past decades, there has been a great deal of research on the use of microorganisms as biological control agents for pests and diseases. More and more bacteria have been identified as biocontrol agents of plant parasitic nematodes with demonstrated inhibitory effects on nematode pest populations (Tian et al., 2010; Migunova and Sasanelli, 2021). Using microorganisms, including antagonistic microorganisms and endophytes, is today an important strategy to control RKNs (Xiang et al., 2018). The search for novel biocontrol often leads to the plant rhizosphere, where the number of bacteria is 100 times higher than in other regions. These bacteria are mainly *Bacillus* and *Pseudomonas* and play a significant role in biocontrol by suppressing RKNs (Mandic-Mulec et al., 2015; Abd-Elgawad, 2016; Dehghanian et al., 2020). Compared with other biological control bacteria, *Bacillus* has outstanding advantages in production, transportation, storage, and application (Lalloo et al., 2010). Many *Bacillus* isolates were verified to have nematicidal activity against RKNs *in vitro* and *in vivo*, such as *Bacillus cereus*, *Bacillus subtilis*, *Bacillus firmus*, and *Bacillus amyloliquefaciens* (Liu et al., 2013; Xiong et al., 2015; Cao et al., 2019; Hu et al., 2020). In recent years, the role of *Bacillus* in microbial application has become increasingly prominent, as *Bacillus* from the plant rhizosphere easily colonizes the roots, both protecting plants from pathogens and promoting plant growth (Sun et al., 2021).

In this study, *Bacillus velezensis* strain Bv-25 was isolated from rhizosphere soil of cucumber (*Cucumis sativus*) cultivar Zhongnong 6 in Langfang city (Hebei, China). The biocontrol activity of strain Bv-25 against *M. incognita* was evaluated. Furthermore, the effect of this treatment on three key activity genes in *M. incognita* and several defense pathway genes of cucumber were measured, to better understand the genetic mechanisms behind the biocontrol effects. Pot tests and field experiments were carried out to verify the control effect. The objective of this study was to evaluate the nematicidal activity of *B. velezensis* strain Bv-25 against *M. incognita* as a potential biocontrol agent and to discover the molecular underpinnings of this effect.

## Materials and Methods

### Source, Isolation of *Bacillus velezensis* and *Meloidogyne incognita*, and Sterilization of Cucumber Seeds

The *M. incognita* used in this study was obtained from the Institute of Vegetables and Flowers, Chinese Academy of Agricultural Sciences (IVF-CAAS; Beijing, China). The second-stage *M. incognita* juveniles (J2s) were inoculated on pepper (*Capsicum annuum* cv. Qiemen) plants and maintained in the greenhouses of the IVF-CAAS for 40 days until egg masses formed. The egg masses were treated with 2% NaOCl and incubated at 28°C for 24 h in sterile water to hatch J2s.

*Bacillus velezensi*s strain Bv-25 was isolated from the rhizosphere soil of cucumber plants. The soil was sampled from a greenhouse in Langfang city (Hebei, China) during the fruiting period (May 2) and used to isolate bacteria using the gradient dilution method on Luria–Bertani (LB) agar medium at 28°C for 48 h. For molecular identification, the 16S ribosomal DNA was amplified by polymerase chain reaction (PCR) with primers 27F and 1492R (Alberoni et al., 2019). The sequence was analyzed using NCBI BLAST^1^ The bacterium was preserved in the Provincial Common Microbe Center of the Chinese Committee for Microbial Culture Conservation (preservation no. CGMCCNO.16523).

Zhongnong no. 6 cucumber seed was obtained from IVF-CAAS. The cucumber seeds were surface-sterilized in 0.5% NaOCl, washed thoroughly with sterile water, and germinated on sterile, moist filter paper in a Petri dish for 3 days at 28°C under darkness. Then, the germinated cucumber seeds were maintained in a sterile mixture of peat:vermiculite (2:1, vol/vol) in a growth chamber with a 15-h light and 9-h dark cycle at 28° for 2 weeks to get cucumber seedlings.

### Nematocidal Efficacy Test *in vitro*

*Bacillus velezensis* strain Bv-25 was cultured for 48 h in 100 mL of LB liquid medium (10 g tryptone, 5 g yeast extract, and 10 g NaCl in 1 L sterilized water) at 28°C on a shaking incubator (200 revolutions/min [rpm]) and adjusted to a density of 1.0 × 10^8^ colony-forming units (CFUs) per milliliter with sterile water. To collect the supernatant, the Bv-25 fermentation broth was centrifuged at 12,000 rpm for 3 min. Each test for nematicidal activity involved six treatments: 10% and 100% fermentation broth, 10% and 100% supernatant from the broth, abamectin (1 μg/mL) as positive control, and sterile water as negative control. To reduce errors, each treatment had 10 replicates, and each test was repeated three times.

The tests were conducted in 24-well plates incubated at 28°C for 24 h. The J2s were considered dead when the bodies of nematodes were straight and did not move upon stimulation with 0.5 M NaOH (Harada and Yoshiga, 2016). Nematode mortality rates in each treatment were recorded at 24 h after treatment. The adjusted mortality rate was calculated by the following formula: [(Mortality rate of nematodes treated by Bv-25 − Mortality rate of nematodes treated by water)/(1 − Mortality rate of nematodes treated by water)] × 100.

### Inhibition of *Meloidogyne incognita* Egg Hatching by Strain Bv-25

To investigate the inhibition effect of strain BV-25 on *M. incognita* egg hatching, we conducted experiments with four treatments: Bv-25 fermentation broth at concentration 1.0 × 10^8^ CFUs/mL, the supernatant, 10% LB medium, and sterilized water as negative control. Each treatment had 10 replicates, and each experiment was repeated three times. A total of 150 eggs were mixed with each treatment in separate wells. The 24-well plates were incubated at 28°C for 72 h in a growth chamber (Mendoza et al., 2008). The numbers of hatched J2s were counted at 72 h after incubation (Saikia et al., 2013). The egg-hatching rate was calculated according to the following formula: [(Number of hatched J2/Total eggs) × 100].

### Effect of Strain Bv-25 Fumigation on the Key Genes of *Meloidogyne incognita* J2s

To understand the molecular effect of Bv-25 volatile compounds on the J2s, we conducted fumigation experiments in three-well culture dishes according to a previously published method (Gu et al., 2007). Five milliliters of either fermentation broth (1.0 × 10^8^ CFUs/mL) or sterile water as a control was put in one well of a dish, and 1 mL of a suspension containing 5,000 J2s added to another, separate well. The dishes were sealed with two layers of sealing film and placed in an incubator at 28°C for 48 h. Three experiments of 15 dishes per treatment were performed. The mortality rate and status of J2s were checked every 24 h by spot checks. The death of J2s was verified as described previously (Harada and Yoshiga, 2016).

Total RNA was extracted from J2s using a tissue cell RNA microextraction kit (Beijing Zhongchuang) every 24 h. The converted first-strand cDNA was obtained using the premix for two-step reverse transcriptase (RT)–quantitative PCR (qPCR)–type reverse transcription reagent (Eric Bio) and subjected to gradient dilution of at least five to six gradients as a template for real-time PCR. qPCR was performed for two essential nematode genes *mpk-1* and *flp-18*, and the chemosensory gene *ord-1*, using the primers in Supplementary Table 1 (Dong et al., 2014, 2016; Shivakumara et al., 2019). The calculation formula for the standard curve was automatically obtained through the BIORAD system: *Y* = *aX* + *b*, where *Y* is the expression amount and *X* is the Ct value (Cottyn et al., 2011). Time at 0 h served as the control.

### Cucumber Root Colonization Test

The roots of cucumber seedlings were immersed in strain Bv-25 fermentation broth (1.0 × 10^8^ CFUs/mL) for 0, 0.5, 1, 2, 3, 6, 9, 12, 24, or 48 h. At each sampled time, the roots of 10 cucumber seedlings were harvested, dried with absorbent paper, cut with sterile scissors, and macerated. Three samples of 0.2 g each were used to extract DNA using the TIANamp DNA Kit (TIANGEN, Beijing, China).

The quantities of Bv-25 on the cucumber roots were determined by real-time PCR. The specific primers were BV-25-F (5′-GTGAGGTAACGGCTCACCAA-3′) and BV-25-R (5′-TGCTCCGTCAGACTTTCGTC-3′), which amplified a 142-bp fragment. Then, real-time PCR SYBR Green I reaction system was prepared in a 20-μL volume, consisting of 10 μL of 2 × Super Real PreMix Plus (TIANGEN), 1 μL each of BV-25-F and BV-25-R primer (0.1 μmol/μL), 1 μL of genomic DNA (diluted 10-fold with double distilled [dd] water) and 7 μL of dd water. The PCR reaction procedure used was as follows: 95°C for 15 min and then 40 cycles of 95°C for 10 s and 60°C for 32 s. From 65 to 95°C, the fluorescence signal was recorded every 0.5°C. The Ct values and regression equation were determined as per a previous method (Cottyn et al., 2011) and used to estimate the quantities of BV-25 on cucumber roots.

To visually observe the infection, cucumber seedlings roots were immersed in Bv-25 fermentation broth (1.0 × 10^8^ CFUs/mL) for 3 h to allow the bacteria to attach to the roots or sterile water as a negative control. Then, the cucumber seedlings were inoculated with approximately 300 J2s in a Petri dish and incubated in a chamber at 28°C for 48 h. To maintain moisture during the incubation period, 3 mL sterile water was added to each Petri dish. After 48 h of incubation, the roots were stained with fuchsin (Bybd et al., 1983) and observed under a microscope.

### Analysis of Defense Signal Pathway Genes Expression in Cucumber Roots

The cucumber seedling roots were soaked in strain Bv-25 fermentation broth and sampled as previously mentioned. Three root samples were collected at each time point, frozen in liquid nitrogen, and stored at −80°C. To analyze gene expression, total RNA extraction from root samples was conducted using the RNAprep Pure Plant Kit (TIANGEN), and cDNA synthesis was performed using the FastKing gDNA Dispelling RT SuperMix (TIANGEN). The expression characteristic of actin (internal control) and four defense signal pathway genes (*pr1*, *pr2*, *lox1*, and *ctr1*) were analyzed by a real-time PCR SYBR Green I reaction system with gene-specific primers (Supplementary Table 1) (Shoresh et al., 2005; Wan et al., 2010; Migocka and Papierniak, 2011). The expression level of the four genes was normalized and compared with the expression level of actin quantified by the ΔΔCt method (Beaubois et al., 2007).

### Split-Root Experiment

To evaluate the induction of resistance against *M. incognita* mediated by strain Bv-25 on cucumber seedlings, we conducted a split-root experiment. The cucumber seedlings at the two-leaf stage were transferred to sterilized peat:vermiculite mixture (2:1, vol/vol) in the split-root setup (Martínez-Medina et al., 2017), consisting of two 500-mL adjacent pot compartments. Forty-eight hours after transplanting, approximately 300 J2s were inoculated to the roots in the pots on the right side (Xing et al., 2017). Ten milliliters of Bv-25 fermentation broth (1.0 × 10^8^ CFUs/mL) or sterile water as negative control was inoculated into the pot on the left side. Three experiments were done with 10 replicates per treatment. The experiments were conducted in a greenhouse under conditions of 28°C and 9-h light/15-h dark. Thirty days after J2s inoculation, the numbers of galls on the cucumber roots treated by Bv-25 and the controls were counted. Control efficiency was calculated according to formula: [(Number of galls on cucumber root under control − Number of galls on cucumber root under Bv-25 treatment)/Number of galls on cucumber root under control] × 100.

### Pot Experiment

The cucumber seedlings at the two-leaf stage were transplanted to pot (three cucumber seedlings per pot) filled with 2,000 mL sterile peat: vermiculite mixture (2:1, vol/vol). The pots were located in the greenhouse under conditions of 28°C and 9-h light/15-h dark. One week after transplanting, an experiment was conducted consisting of three treatments: Bv-25 fermentation broth (1.0 × 10^8^ CFUs/mL), a 1/1,000 dilution of 1.8% avermectin (Green Agricultural Science and Technology Group, Beijing), and sterile water as control. A total of 300 J2s were inoculated to each pot. Three such experiments of 10 replicates per treatment were performed. Galls on cucumber roots were counted at 30 days after J2 inoculation. The control efficiency was calculated as before.

### Field Experiment

Field experiments were performed in a greenhouse with heavy *M. incognita* infestation in Langfang City (Hebei, China) in April 2019. The greenhouse was divided into nine blocks (9 m^2^) arranged using randomized block design with three replications. Each block consisted of 2 rows and 15 cucumber plants planted per row. The row space was 50 cm and plant-to-plant spacing was 40 cm. Before cucumber seedling transplantation, the population densities of *M. incognita* were counted using the method of Yin et al. (2021).

Cucumber seedlings (cultivar Zhongnong 6) at the two-leaf stage were transplanted into the greenhouse. For Bv-25 treatment, each cucumber seedling was irrigated with a mixture of 100 mL of Bv-25 fermentation broth (1.0 × 10^8^ CFUs/mL) and 400 mL of water. In the nematicide control, 10% fosthiazate granules (Ishihara Industrial Co., Ltd., Japan) were applied to the soil at the depth of 15 to 20 cm in line with the state standards (22.5 kg/ha) before transplanting. The untreated control was irrigated with water. Each treatment had three replicates. The shoot length of the cucumber plants and fruit weight were measured three times. Regular management was carried out in the greenhouse during the experimental period. The gall index was scored on a 1- to 5-point scale: 1 = no galls; 2 = 1–25% galled roots; 3 = 26–50% galled roots; 4 = 51–75% galled roots; 5 = > 75% galled roots (Dube and Smart, 1987). The disease index was calculated according to the following formula: Disease index = [Σ (Number of cucumbers in the gall index × Gall index)/(Number of total cucumbers × Highest gall index)] × 100. The managing effect of strain Bv-25 against *M. incognita* in the field was assessed with the formula: Managing effect (%) = [(Disease index of control − Disease index of Bv-25 treatment)/Disease index of control] × 100.

### Statistical Analysis

The statistical analyses were performed using one-way analysis of variance (ANOVA) with Duncan multiple-range test (*p* < 0.05) and Tukey multiple-comparisons test (*p* < 0.001) (SPSS software, v.220; SPSS Inc., Chicago, IL, United States). Regression analysis of Bv-25 quantification was also carried out with SPSS. All data are presented as mean ± standard error.

## Results

### Nematicidal Activity of Strain Bv-25 Against *Meloidogyne incognita*

**FIGURE 1:**
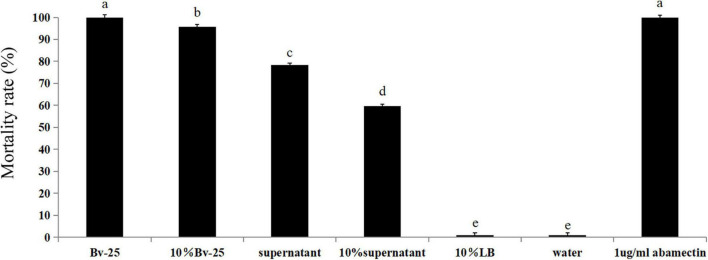
Mortality rate of J2 treated by *B. velezensis* strain Bv-25 *in vitro*. Mortality rates were assessed by 300 J2s immersed in Bv-25 fermentation broth, 10% fermentation broth, bacteria-free supernatant, and 10% supernatant for 24 h at 28°C. Abamectin (1 μg/mL) was a positive control, whereas sterile water served as negative control. Different letters indicate significant differences (*p* < 0.05; *n* = 30) between treatments according to Duncan multiple-range test following one-way ANOVA.

The mortality rates of *M. incognita* after treatment with abamectin, the Bv-25 fermentation broth, and 10% fermentation broth were 100%, 100%, and 96.1%, respectively, which were significantly higher than those of other treatments (Figure 1). The mortality rates for the supernatant broth and 10% supernatant were 78.3% and 59.5%, respectively (Figure 1). The mortality rates for water and 10% LB medium were the lowest. Strain Bv-25 thus had strong nematicidal activity against *M. incognita*.

### Effect of Strain Bv-25 on the Eggs Hatching of *Meloidogyne incognita*

**FIGURE 2:**
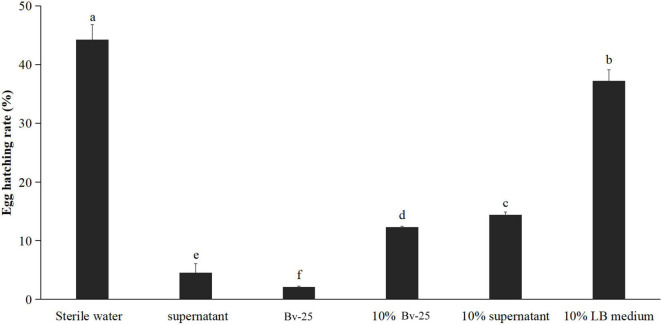
Strain Bv-25 inhibited the hatching of *M. incognita* eggs *in vitro*. Egg-hatching rates were evaluated by treating 150 eggs with the Bv-25 fermentation broth, 10% fermentation broth, supernatant, and 10% supernatant for 72 h at 28°C. The negative control was sterile water. Different letters indicate significant differences (*p* < 0.05; n = 30) between treatments according to Duncan multiple-range test following one-way ANOVA.

The egg-hatching rate of *M. incognita* eggs 72 h after treatment with strain Bv-25 fermentation broth was the lowest (2.1%), followed by the Bv-25 supernatant (4.6%), 10% fermentation broth (12.3%), 10% supernatant (14.4%), 10% LB medium (37.2%), and sterile water (44.2%) (Figure 2). Bv-25 treatment thus markedly suppressed egg hatching of *M. incognita*.

### Effect of Strain Bv-25 Fumigation on the Key Genes of *Meloidogyne incognita* J2s

**FIGURE 3:**
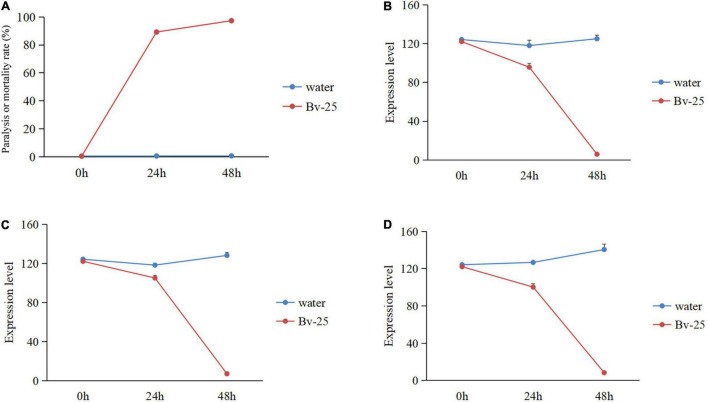
The nematicidal activity and gene expression pattern of J2s under Bv-25 fumigation. **(A)** The paralysis or mortality rate of J2s treated by Bv-25 fumigation. **(B)** The *ord-1* gene expression pattern. **(C)** The *mpk-1* gene expression pattern. **(D)** The *flp-1*8 gene expression pattern.

Under Bv-25 fumigation treatment, most J2s (89.2%) gradually reduced movement within 24 h but recovered mobility with NaOH treatment, suggesting that the J2s were paralyzed by volatile organic compounds produced by Bv-25 within 24 h. At 48 h, nearly all J2s (98.2%) could not move in response to NaOH and were regarded as dead. The paralysis or mortality rates of J2s treated with Bv-25 fumigation were much higher than the water controls (Figure 3A).

To further assess the effect of Bv-25 fumigation on J2s, three key *M. incognita* genes were further analyzed by qPCR. The results show that a good linear relationship existed between the Ct value of the three-gene amplification reaction cycle numbers and the logarithmic value of the DNA copy number (*flp-18*: *y* = 4.945*x* + 17.865, *R*^2^ = 0.9994; *ord-1*: *y* = 3.2051*x* + 22.681, *R*^2^ = 0.9964. *mpk-1*: *y* = 3.5399*x* + 19.066, *R*^2^ = 0.9944). The expression patterns of the three genes were significantly different between the water control and the Bv-25 fumigation treatment, being relatively steady with water but decreasing sharply with Bv-25 fumigation from 0 to 48 h. The expression of *ord-1* decreased 122.1 times in the treated nematodes compared with control (Figure 3B), *mpk-1* decreased 67.8 times from 122.1 to 1.8 (Figure 3C), and *flp-1*8 decreased 32.1 times from 122.1 to 3.8 (Figure 3D).

### Colonization on Cucumber Roots and Suppression of *Meloidogyne incognita* Infection by Strain Bv-25

The Ct value of the amplification reaction cycle had a linear relationship with the logarithmic value of the Bv-25 concentration (*y* = −3.03x + 34.968; *R*^2^ = 0.9909; *p* < 0.01). The Ct value decreased with increasing time, demonstrating an increase in Bv-25 colonization on cucumber roots (Table 1). From 24 to 36 h, the concentration of Bv-25 markedly increased from 5.94 × 10^7^ CFUs/g to 3.26 × 10^8^ CFUs/g, suggesting that strain Bv-25 was able to colonize cucumber roots and reproduce rapidly.

**TABLE 1:** Quantification of strain Bv-25 on cucumber roots by quantitative PCR.

Treatment time (h)	Ct value (0.2 g roots)	Quantity of Bv-25 on cucumber roots (CFU/g)
0 h	>28	–
0.5 h	21.55	1.34 × 10^5^
1 h	19.83	4.95 × 10^5^
2 h	19.40	6.86 × 10^5^
3 h 6 h	18.21 17.29	1.69 × 10^6^ 3.41 × 10^6^
9 h	16.45	6.64 × 10^6^
12 h	14.42	3.02 × 10^6^
24 h 36 h 48 h	13.53 11.29 9.29	5.94 × 10^7^ 3.26 × 10^8^ 1.49 × 10^8^

**FIGURE 4:**
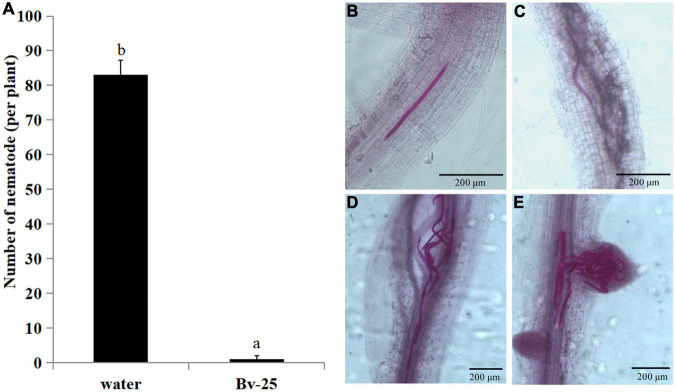
Effect of strain Bv-25 on *M. incognita* infection *in vitro*. **(A)** The number of J2s entered into roots treated with Bv-25 or sterile water. Different letters indicate significant differences (*p* < 0.05; n = 30) between treatments according to Duncan multiple-range test following one-way ANOVA. **(B–E)** J2s in cucumber root treated by Bv-25 **(B,C)** and sterile water **(D,E)**. The roots were stained with acid fuchsin. Live nematodes appear bright pink. Scale bars = 50 μm **(C)** and 200 μm.

Forty-eight hours after inoculation, the number of J2s in cucumber roots treated by strain Bv-25 was 4.3 per plant and markedly less than that in control roots with 83.0 (Figure 4A). Under control treatment, plenty of J2s entered into the vascular bundles and grouped together (Figures 4B–E). The results indicate that strain Bv-25 could inhibit *M. incognita* invasion into cucumber roots.

### Effect of Strain Bv-25 on the Defense Genes and Induced Resistance of Cucumber

**FIGURE 5:**
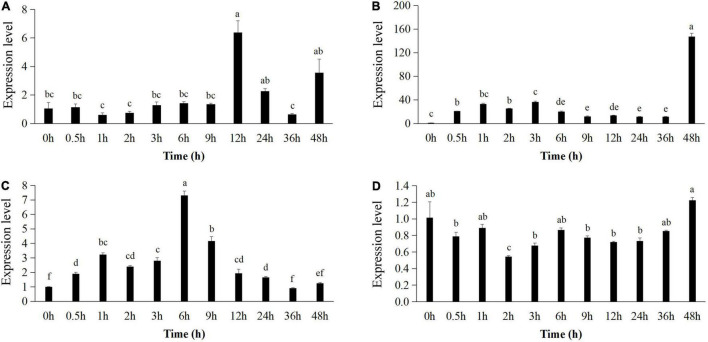
Expression of genes related to defense responses in cucumber roots. **(A–D)** Expression level of *pr1* **(A)**, *pr2* **(B)**, *lox1* **(C)**, and *ctr1* **(D)** in cucumber roots treated by Bv-25 or sterile water. Different letters indicate significant differences (*p* < 0.05; n = 9) between treatments according to Duncan multiple-range test following one-way ANOVA.

**FIGURE 6:**
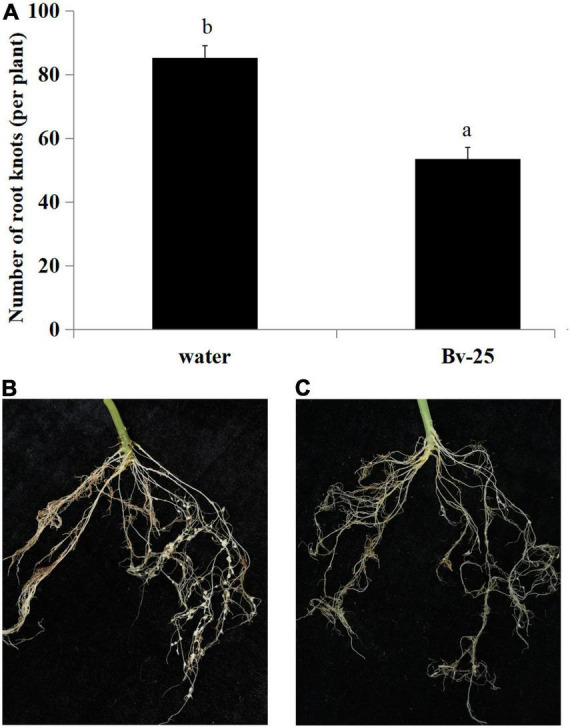
Suppression of *M. incognita* infection by strain Bv-25. **(A)** Number of root galls on cucumber seedlings treated with Bv-25 or water. **(B)** Root galls on cucumber seedling treated with water. **(C)** Root galls on cucumber seedling treated with Bv-25. Different letters indicate significant differences (*p* < 0.05; *n* = 30) between treatments according to Duncan multiple-range test following one-way ANOVA.

The expression pattern of *pr1* was significantly up-regulated, reaching the highest level at 12 h (Figure 5A), whereas *pr3* reached the highest expression level at 48 h, 140 times higher than that of the control group (Figure 5B). The expression of *lox1* increased at first and then decreased after 1 h, increased and reached the peak at 6 h (Figure 5C), and then declined until 48 h. There was no significant difference in *ctr1* gene expression between control and Bv-25–treated group (Figure 5D). These results indicated that strain Bv-25 activates the salicylic acid (SA) and jasmonic acid (JA) defense signaling pathways in cucumber roots. In the split-root experiment, the average number of root galls on cucumber roots treated by strain Bv-25 (53.5 per plant) was significantly lower than that on control roots (85.3 per plant) (Figures 6A–C). The results together showed that Bv-25 could enhance resistance of cucumber to reduce *M. incognita* infection.

### Control Effect of Strain Bv-25 Against *Meloidogyne incognita* in Pot and Field Experiments

**FIGURE 7:**
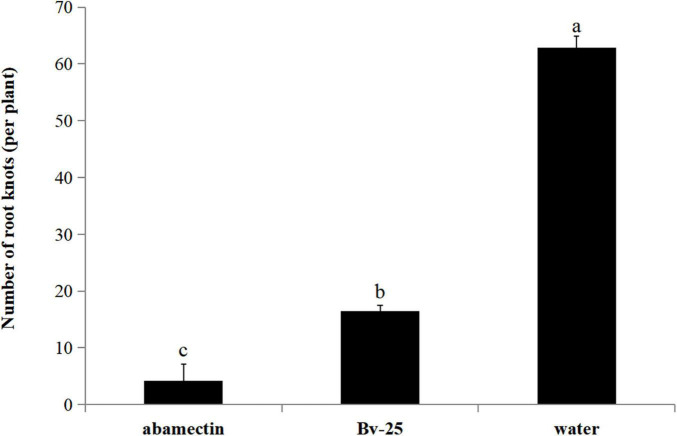
Control efficiency of Bv-25 on *M. incognita* in the pot experiment. Cucumber seedlings were treated by Bv-25 fermentation broth, 1.8% avermectin, or sterile water. A total of 300 *M. incognita* J2s were inoculated to the cucumber roots. The number of galls was counted after 30 days. Different letters indicate significant differences (*p* < 0.05; *n* = 30) between treatments according to Duncan multiple-range test following one-way ANOVA.

In the pot experiment, cucumber roots treated with strain Bv-25 fermentation broth and abamectin had fewer root nodes (4.2 and 16.4, respectively) than those treated with water (62.8) (Figure 7). For strain Bv-25, this amounted to a 73.8% decrease in the number of cucumber root nodes.

The soil in the greenhouse had many *M. incognita* (43 J2s/100 g of soil). Cucumber plants in the control plots had significantly more root galls, shorter heights, and lower yields than treated plants. The disease index was markedly higher in control plants (88.4) than in the Bv-25 treatment (33.9) and fosthiazate treatment (23.4) plants. The control effect of Bv-25 treatment was 61.6%, and it increased heights and yielded 14.4 and 36.5%, respectively (Table 2).

**TABLE 2:** Control effect of strain Bv-25 in greenhouse.

Treatment	Plant height (cm)			Yield (kg/polt)				Disease index
	2019.05.12	2019.05.20	2019.05.27	2019.05.20	2019.05.27	2019.06.03	total	2019.06.10
								
10% Fosthiazate	72.3 ± 1.8b	144.3 ± 2.3b	206.3 ± 0.7a	0.00 ± 0.00a	1.15 ± 0.24a	11.28 ± 0.85a	12.42 ± 1.09b	23.4 ± 2.06a
Bv-25	84.8 ± 1.8c	151.7 ± 2.4c	220.2 ± 4.6b	1.92 ± 0.21b	2.26 ± 0.31b	7.48 ± 0.85b	11.67 ± 1.11b	33.9 ± 1.25a
Control	62.2 ± 1.1a	133.6 ± 0.4a	203.2 ± 0.6a	0.00 ± 0.00a	0.91 ± 0.28a	7.63 ± 1.26b	8.55 ± 0.98a	88.4 ± 4.15b

*Data represented mean ± standard error of three independent biological and ten technical replicates (n = 30) and was analyzed by one-way ANOVA with Duncan’s multiple range test. Different letters indicate significant differences (P < 0.05).*

## Discussion

In this study, a novel *B. velezensis* strain Bv-25 was isolated from the rhizosphere of cucumber plants. The strain could efficiently kill most *M. incognita* J2s and suppress eggs hatching *in vitro* and was found to inhibit key genes of the nematode. Bv-25 successfully colonized cucumber roots and then induced resistance against *M. incognita* infection in part by up-regulating plant defense genes. The pot and field tests demonstrated that Bv-25 could effectively control *M. incognita*, inhibiting root knot formation, promoting plant growth, and increasing yields. It thus has great potential as a biocontrol agent against *M. incognita*.

To more deeply reveal the mechanism for control, we analyzed the effect of Bv-25 treatment on the key genes of *M. incognita* J2s, such as *flp-18*, *mpk-1*, and *odr-1*. FMRFamide-like peptides encoded by *flp* genes play diverse roles in nematode physiological activity, such as locomotion, feeding, and reproduction (Johnston et al., 2010). Transgenic rice that effectively decreased *flp-1* and *flp-12* expression in *Meloidogyne graminicola* by the host-induced gene silencing method significantly inhibited nematode infection and oviposition (Hada et al., 2020). The mitogen-activated protein kinase encoded by *mpk-1* is an important transcriptional regulatory factor and protein kinase (Okuyama et al., 2010). The RNA interference technique has been used to down-regulate the transcription of *flp-18* and *mpk-1* genes in *M. incognita* and suppress their infection (Dong et al., 2016). In our study, Bv-25 significantly inhibited the expression of *mpk-1* and *flp-18*, thus killing the J2s.

*Odr-1* is a chemosensory gene with a role in host recognition, enabling nematodes to sense volatile compounds and host root exudates (Shivakumara et al., 2019). Bv-25 treatment suppressed *odr-1* gene expression in J2s 122 fold, suggesting some volatile organic compound(s) produced by Bv-25 impacts the chemosensory function of *M. incognita*. After 48 h of fumigation treatment, the J2s were dead, and the expression of three functional genes also reduced to minimum. The data suggest Bv-25 can be used to control *M. incognita* through fumigation, which is similar to how the bacterium *Pseudomonas putida* 1A00316 produces 2-undecanone that acts as a fumigant against *M. incognita* (Zhai et al., 2018). Future studies should further isolate the volatile organic compounds with nematicidal activity from Bv-25.

Effective colonization of the root system is important for biocontrol microbes against root pathogens (Weng et al., 2013). Some microorganisms can survive in the biofilm on plant root surfaces (Davey and O’toole, 2000; O’Toole et al., 2000), shaping the characteristics of root metabolite production and secretion, thus promoting host resistance and suppressing nematode invasion (Hashem and Abo-Elyousr, 2011). Arbuscular mycorrhizal fungi efficiently colonize roots of *Cymbopogon citratus* and induce the activity of defense-related enzymes, such as peroxidase, β-1,3-glucanase, and polyphenol oxidase, thus suppressing root lesion nematode infection (Silva et al., 2021). *Arabidopsis thaliana* root colonization by *B. firmus* I-1582 significantly inhibits the infection and reproduction of the beet cyst nematode *Heterodera schachtii* (Huang et al., 2021). In our study, the Bv-25 was obtained from the rhizosphere of cucumber and so easily colonized cucumber roots. Quantitative experiments showed that the number of bacteria on cucumber roots reached 5.94 × 10^7^ CFUs/g after inoculation for 36 h. Thus, strain Bv-25 colonized cucumber roots rapidly and in large quantities while suppressing *M. incognita*, which is similar to *B. cereus* strain Bc-cm103 obtained from *Cucumis metuliferus* (Yin et al., 2021).

RKN invasion will quickly trigger plant defense responses once they are induced by biocontrol bacteria (Vos et al., 2013). Biocontrol bacteria thus can improve the activity of biomolecules and enzymes relevant to antinematode defense (Vos et al., 2013). In this study, the split-root experiment demonstrated that strain Bv-25 could induce cucumber root resistance against *M. incognita*, and the qPCR assay confirmed that it activated multiple defenses in cucumber seedlings. In general, the SA, JA, or ethylene signaling pathways regulate the major plant defenses (Shoresh et al., 2005). The *pr1* and *pr3* genes play important roles in the SA signaling pathway (Islam and Babadoost, 2010), which some biocontrol microbes induce. For example, *Piriformospora indica* can promote the expression of *pr1* and *pr3* genes in cucumber and enhance resistance to *M. incognita* (Atia et al., 2020). The biocontrol fungus *Trichoderma harzianum* T-78 up-regulates the expression of *pr1a* and *pr6a* in tomato and inhibits *M. incognita* infection (Martínez-Medina et al., 2017). In our study, the relative expression level of *pr3* in cucumber root reached its peak after 48-h treatment with Bv-25, whereas the expression level of *pr1* peaked at 12 h. The increased expression of two *pr* genes indicates that Bv-25 activated the defense response of cucumber roots to *M. incognita* infection through the SA signaling pathway, which is in line with the results by Atia et al. (2020) and Martínez-Medina et al. (2017).

The *lox1* gene, a key gene in JA biosynthesis (Hornung et al., 1999), is activated by cyst nematode infection, and more LOX proteins were expressed in resistant roots compared with susceptible roots (Leone et al., 2001). The biocontrol bacterium *B. cereus* strain Bc-cm103 increases the expression of *lox1* in cucumber roots and suppresses infection with *M. incognita* (Yin et al., 2021). Similarly, in our study, Bv-25 enhanced the expression of *lox1* of cucumber roots at 6 h, thus promoting resistance to *M. incognita*.

The *ctr1* gene encodes a negative regulator in ethylene signaling when ethylene is lacking (Huang et al., 2003). We found no significant difference in *ctr1* gene expression between control and Bv-25–treated plants, suggesting that the bacterium did not regulate the ethylene signaling in cucumber. Similarly, the *ctr1* gene of *Arabidopsis* had no measurable effect on plant susceptibility to *H. schachtii* (Piya et al., 2019).

In conclusion, the strain Bv-25 exhibited direct nematicidal against *M. incognita in vitro*, suppressed gene expression of *M. incognita* J2s as a fumigant, and remarkably reduced nematode infection in laboratory trials. Strain Bv-25 also promoted gene expression in the SA and JA signaling pathways, consequently improving cucumber resistance against *M. incognita*. In pot and field trials, strain Bv-25 significantly decreased the disease index of *M. incognita* and increased cucumber yield. On the whole, strain Bv-25 has multiple antinematode properties that justify its application in biocontrol against *M. incognita*.

## Data Availability Statement

The original contributions presented in the study are included in the article/Supplementary Material, further inquiries can be directed to the corresponding author/s.

## Author Contributions

X-LT and X-MZ: conceptualization and writing—original draft preparation. X-MZ and S-YZ: methodology and investigation. X-LT: formal analysis. S-YZ: resources. J-LZ, and Z-CM: writing—review and editing. Z-CM: supervision. X-LT and Z-CM: funding acquisition. All authors contributed to the article and approved the submitted version.

## Conflict of Interest

The authors declare that the research was conducted in the absence of any commercial or financial relationships that could be construed as a potential conflict of interest.

## Publisher’s Note

All claims expressed in this article are solely those of the authors and do not necessarily represent those of their affiliated organizations, or those of the publisher, the editors and the reviewers. Any product that may be evaluated in this article, or claim that may be made by its manufacturer, is not guaranteed or endorsed by the publisher.

## Footnotes

1
http://www.ncbi.nlm.nih.gov/
