# Supplementary material for: The Biocontrol Functions of Bacillus velezensis Strain Bv-25 Against Meloidogyne incognita
Source: Front Microbiol. 2022 Apr 7;13:843041. doi: 10.3389/fmicb.2022.843041 (PMC9022661; doi:10.3389/fmicb.2022.843041)
Supplement: Supplementary file 1 [file Table_1.docx]

**Supplementary TABLE 1 ︱**Detection of nematode active gene primers

| Gene | Primer | primer Sequence | Accession Number |
| --- | --- | --- | --- |
| *flp-18* | Re-flp-18-F | 5'-CCCAAGTTTGAGGGATATT-3′ | AY729022 |
|  | Re-flp-18-R | 5'-ATTATTATGACCCGCCTCT-3′ |  |
| *mpk-1* | Re-mpk-1-F | 5'-GCAGCATTCTCAACAACCAC -3 | DQ923592 |
|  | Re-mpk-1-R | 5'-CAACCATTCCATAAGCACCT-3′ |  |
| *ord-1* | Mi-ord-1-F | 5'-TAGCCCAAGCTCGAGAACAA-3′ | MG780832 |
|  | Mi-ord-1-R | 5'-TAGCGGAATCGTAAGAGCGT -3′ |  |
