# Supplementary material for: The Biocontrol Functions of Bacillus velezensis Strain Bv-25 Against Meloidogyne incognita
Source: Front Microbiol. 2022 Apr 7;13:843041. doi: 10.3389/fmicb.2022.843041 (PMC9022661; doi:10.3389/fmicb.2022.843041)
Supplement: Supplementary file 2 [file Table_2.docx]

**Supplementary TABLE 2︱**Gene primers for induced resistance test

| Gene | Sequence primer | Amplified fragment（bp) |
| --- | --- | --- |
|  |  |  |
| *Actin* | 5’-TCCACGAGACTACCTACAACTC-3’ | 122 bp |
|  | 5’-GCTCATACGGTCAGCGAT-3’ |  |
| *pr1* | 5’-TGCTCAACAAT A TGCGAACC-3’ | 200 bp |
|  | 5’-TCATCCACCCACAACTGAAC-3’ |  |
| *pr3* | 5′-TGGTCACTGCAACCCTGACA -3′ | 200bp |
|  | 5′-AGTGGCCTGGAATCCGACT-3′ |  |
| *lox1* | 5’-AAGGTTTGCCTGTCCCAAGA-3’ | 200 bp |
|  | 5’-TGAGTACTGGATTAACTCCAGCCAA-3’ |  |
| *ctr1* | 5’-AAACACGTCGGA TAAA TA TGGCTT-3’ | 200 bp |
|  | 5’-CATCCATTCAGGCGTTCCAG-3’ |  |
